# A Local Heating Profile to Manage Lower Back Pain in an Automotive Seat: A Pilot Study

**DOI:** 10.3390/bioengineering11101040

**Published:** 2024-10-18

**Authors:** Matt M. Mallette, Nathaniel Gur-Arie, Nicola Gerrett

**Affiliations:** Integrative Human Research Lab, Gentherm, 38455 Hills Tech Dr., Farmington Hills, MI 48331, USA; matt.mallette@gentherm.com (M.M.M.); nathaniel.gur-arie@gentherm.com (N.G.-A.)

**Keywords:** lower back pain, thermal therapy, local heating, automotive seating, thermal comfort, musculoskeletal pain, non-pharmacological pain management

## Abstract

Lower back pain (LBP) is one of the most prevalent health losses in adults worldwide. Historically, heat has been successfully used for treating pain and relieving tight muscles. Given the effective contact with the occupant’s back and proximity to the heat source, coupled with increasing commute times, automotive seats offer an opportunity to intervene. Fifteen adults (nine female) who experienced acute, subacute, and chronic lower back pain were recruited to examine the effectiveness of heat delivered to the lower back in providing temporary pain relief. Participants sat in a car seat for 38 min on two days, which included a 5-min baseline followed by a 33-min intervention; control, or localized. For the control condition, participants sat for 33 min without any thermal devices on, while the localized condition heated and maintained the seat surface temperature of the lower seat back area to ~45 °C. Over the 33-min control condition, the back skin temperature increased by ~1–2 °C and did not impact the subjective LBP. Heating the lower back for 33 min to ~39 °C reduced the subjective LBP by 10%. We demonstrated that lower back pain can be alleviated from an automotive seat providing heat to the lower back within normal commute times in those with lower back pain.

## 1. Introduction

Statistics from the Global Burden of Disease Study (2021) indicate that 619 million people suffer from lower back pain (LBP) at any given time [1]. Lower back pain is one of the leading causes of health loss and disability around the world [1,2]. It impacts both sexes, but the prevalence is higher in females and increases for both sexes with aging. In 2016, lower back pain and neck pain was the most costly health expense in the United States of America, totaling ~USD 134 billion [3]. It has profound societal and economic impacts on the individual, such as reduced productivity, challenged relationships, poor mental health, and reduced quality of life. Furthermore, due to increasing risk factors such as poor workplace ergonomics and body mass index, the prevalence of LBP is expected to rise to 843 million people by 2050 [1].

Pharmacological acute treatment options, such as non-steroidal anti-inflammatory drugs (NSAIDs) and opioids, are often prescribed for LBP treatment, but a systematic review reported small treatment effects, alongside adverse side effects [4]. Several global institutions have proposed non-pharmacological care as a first-line treatment option and reserve pharmacological care as a last resort, to reduce unwanted side effects and polypharmacy [5]. Indeed, several non-pharmacological treatment options for LBP have been explored with varying success, such as heating, cooling, hydrotherapy, transcutaneous electrical nerve stimulation, and acupuncture, among others [6].

A frequently used acute non-pharmacological treatment option for chronic LBP is local heating [7]. Local heating has been used for treating long-term pain and discomfort complaints and can improve quality of life for issues of chronic musculoskeletal pain or pain from menstrual cramps [8,9]. Applying an external heat source increases local skin temperature, which increases local blood flow and activates thermoreceptors. Stimulating the thermoreceptors can reduce sympathetic tone, promoting further smooth muscle relaxation and increasing local blood flow [6]. It is also hypothesized that activating warm sensitive C-nerve fibers [10] and Transient Receptor Potential Vanilloid-1 (TRPV-1) receptors at temperatures greater than 43 °C results in the de-activation of noxious receptors on the same free nerve endings [11,12], which is why heating has an analgesic effect—often called the gate control theory [13]. The gate control theory of pain refers to a mechanism whereby non-painful sensory signals, such as pressure or temperature, can block pain signals from reaching the brain, thereby reducing the pain perceived [13]. Despite heating being used frequently to manage LBP, there is limited evidence of effective temperatures or a dose-response curve, particularly concerning short treatment windows, such as the typical commute time.

External heating modalities exist in various forms, but the ease of access and simplicity of use are preferred for adherence and impact. For sustained usage, the heating method must seamlessly integrate into users’ daily routines, minimizing the need for additional effort. Chemical heat wraps (40 °C applied directly to the lumbar region) worn for 8 h have demonstrated a greater impact on reducing LBP than NSAIDs [14]. Alternatively, higher temperatures for a shorter duration can also have beneficial impacts on pain. Increasing the temperature of a small adhesive metal disk (~5 cm^2^) from 40 °C to 45 °C for 10 s in ‘pulses’ for 30 min reduced pain more than 30 min of constant 37 °C [7]. Skin temperatures above 40 °C appear to be common in the literature for alleviating LBP [7,14,15]. To maximize thermal analgesic effects, skin temperatures of ~43 °C would activate TRPV-1 receptors, whereas temperatures below 43 °C would take advantage of increased local blood flow and gate control theory.

While these two therapies, above [7,14], highlight two different methods of applying heat energy to the body to alleviate LBP (high heat over a short period compared to lower heat over a longer time), both of these modalities are applied directly to the skin, a method that cannot be achieved in all applications. Anytime a medium exists between the heat source and the target (e.g., the skin), some of that heat energy will be lost due to the insulative qualities of the medium. In an automotive application, those mediums between the heating element and the skin typically are clothing (t-shirt, thermal resistance (*R*) = 0.02325 m^2^·K/W; sweater *R* = 0.0527 m^2^·K/W; jacket, *R* = 0.10075 m^2^·K/W, etc.) and all of the layers of material between the heating element and the seat’s surface—such as a foam liner, layers of polyester trim, and/or leather (cloth seat, thermal conductivity (k) = 0.0359 W/m·k; or an unperforated leather seat, k = 0.1467 W/m·k). In addition to the insulation between the heating element and the skin, the material in the seat behind the heater wire (e.g., a large foam pad) will absorb heat energy as well, reducing the amount of heat energy delivered to the skin. These factors create a situation where the temperature of the heating element will always be higher (>10 °C) than the skin temperature. Finally, another challenge of providing therapeutic heat in an automotive seat is that the heating elements are fixed in position after production; therefore, differences in body morphology and sitting posture may impact the heat delivered to the occupant [16]. In the case of low back pain, due to the forced posture of the buttocks, sacral, and lumbar region, heat should be able to be delivered to the lower back well for a wide variety of body shapes and sizes; however, individual differences will occur.

Heated technologies have existed in automotive vehicles (e.g., heated seats and heated steering wheels) for over 50 years and there is anecdotal evidence of people using these features to manage pain, despite their primary function being for thermal comfort [17]. In the US, car ownership is at 86% and American drivers spend 17,600 min/year behind the wheel, with an average all-purpose drive time of 19 min [18]. Additionally, in the US, work commute times are increasing post-COVID-19 pandemic, and in 2022 reached an average of ~26.5 min [19], offering ample time to provide therapeutic heat in a low-effort manner, directly to the occupant’s back, when the occupant is ‘forced’ to remain relatively still during the drive.

In addition to providing thermal comfort to occupants, automakers have recently been focusing on creating a thermal microclimate enveloping each occupant as opposed to conditioning the entire cabin due to the energy-saving potential for electric vehicles [20]. Because the thermal technology in these seats has been developed for thermal comfort and not pain management, there are no temperature profiles to manage lower back pain within the automotive space. This investigation aims to evaluate if local heating provided to the lower back in a temperature-controlled automotive seat can reduce pain perception in a population with lower back pain and discomfort complaints within typical commute times.

## 2. Materials and Methods

### 2.1. Participants

Fifteen participants (six male, nine female, age 45 ± 13 yrs, height 167 ± 10 cm, weight 98.9 ± 20.8 kg) were recruited through an external recruitment agency. Participants were paid USD 450 for the completion of the study. Participants were included if they were currently experiencing non-specific acute (*n* = 5), subacute (*n* = 5), or chronic back pain (*n* = 5). Acute back pain was defined as pain lasting less than 4 weeks, subacute was defined as lower back pain lasting 4–12 weeks, and chronic back pain as lasting more than 12 weeks. Participants were excluded if they had any other underlying health conditions including any cardiovascular conditions, sensory impairments, or type I or type II diabetes.

### 2.2. Research Design

We employed a repeated-measures research design to evaluate two conditions, control and localized therapy in a randomized order, within one week of each other. The different conditions refer to the temperature profiles created using the thermal technology within the seat. For the ‘control’ condition, the thermal actuators in the seat remained off. For the ‘localized’ therapy condition, the thermal technology in the lower third of the seat back was increased to a surface temperature of ~45 °C and held constant for 33 min. Each condition began with a 5-min baseline period. A specialized, high-density zonal heating element was used to heat only the lower third of the seat back. Standard automotive seat heating elements typically have a power density of ~300–800 W/m^2^, whereas the heating element used in this study was between 1100–2500 W/m^2^.

To limit variability in clothing insulation from participants, participants were asked to wear a 100% cotton t-shirt for testing and to wear either the same shirt or a shirt of similar thickness for the subsequent test. The desired skin temperature was determined by benchmarking against a Food and Drug Administration-approved medical device to provide localized conductive heat therapy (Micro-Temp^®^ LT, Gentherm, Cincinnati, OH, USA). This was done by setting the device to its maximum temperature and assessing the skin temperature directly under the heating pad. Then, we determined what seat surface temperature was needed to create the desired skin temperature through pilot work, ensuring that there was no risk of thermal injury at these set points.

### 2.3. Study Protocol

After a telephone screening session, eligible participants were invited to visit the laboratory twice at the same time of day, within one week of each other. On the first visit, they completed a confirmatory health screen questionnaire, were given an overview of the research project, and then provided verbal and written informed consent (Advarra, Columbia, MD, USA, Pro00069393). For each visit, participants were instructed to abstain from any NSAIDs or over-the-counter pain medication, topical creams, cannabinoids, or holistic medicines on the day of each test, but were permitted to take any prescribed medication. Participants were instructed to wear a 100% cotton T-shirt of similar thickness for all tests, to ensure a similar clothing insulation and heat transfer to the occupant’s back for all tests.

On each visit, participants had 9 T-Type thermocouples (Temprel, Cincinnati, OH, USA) affixed using tape (Cover-Roll Stretch, BSN Medical GmbH, Hamburg, Germany) to their skin. The 9 thermocouples were located with 3 across the upper back, 3 across the mid back, and 3 across the lower back. Additionally, 2 thermocouples were attached to the lower third, middle third, and upper third of the seat back, as well as the bolster (side) area of the seat back. For these sensors to equilibrate to body temperature, the participant sat in the seat for ~5 min before the test began. After the sensors reached equilibrium, the 38-min test started, composed of a 5-min baseline period, and a 33-min intervention.

**Figure 1:**
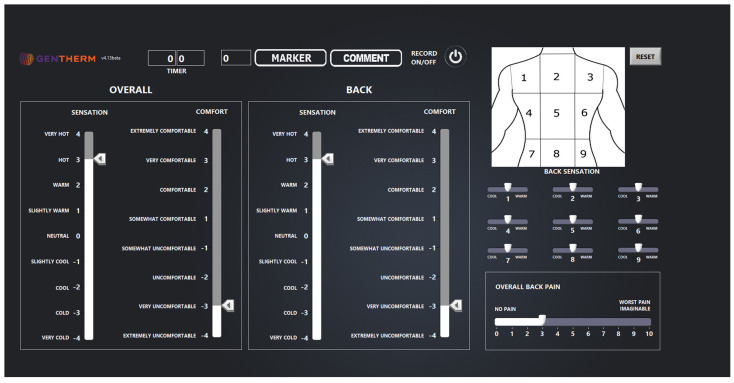
A visual display of the subjective tablet screen where participants continuously recorded their subjective responses. They recorded their overall thermal sensation and overall thermal comfort (**left**), and their back thermal sensation and back thermal comfort (**middle**). They also recorded on a 9-grid matrix if certain areas of their back felt neutral, cool, or warm (**upper right**) and their overall back pain (**bottom right**). They could change their response freely and changes were made by moving the toggle on each scale.

Alongside the measurement of skin and seat surface temperature during the test, participants were also asked to continuously report their subjective thermal comfort, thermal sensation, and pain responses on a tablet located directly in front of them. The subjective application interface is shown in Figure 1. They were instructed to record their overall thermal sensation and thermal comfort, as well as their local back thermal sensation and thermal comfort. The thermal comfort and sensation scales are based on the ISO 10,551 scales [21,22]. Before each test, participants were instructed to make changes to their subjective ratings whenever they felt the need, and were reminded that thermal comfort refers only to how comfortable the temperature of their back is, and to try to limit the impact of their back pain or the physical comfort of the seat on these measures. Also, on a 3 × 3 grid matrix of their back (see upper right section of Figure 1), participants were asked to rate if each grid on their back felt ‘cool’, ‘neutral’, or ‘warm’, in addition to their overall back pain on a 0 to 10 scale, where 0 and 10 were anchored with “No Pain” and “Worst Pain Imaginable”, respectively [23]. Participants could change their response freely and changes were made by moving the toggle on each scale. The subjective application was created using LabView (v.2021 SP1, National Instruments, Austin, TX, USA) and the data was continuously recorded at 10 s intervals.

### 2.4. Data Analysis

Data were synchronized, formatted, visualized, and analyzed using a custom script in Python (v.3.12.0). The nine thermocouples used to measure skin temperatures across the back were averaged for each ‘level’ at the upper, middle, and lower back. The seat surface temperatures were also averaged in four zones; upper, middle, lower, and bolster area. Thermocouple data were collected via an Agilent data acquisition system (Agilent Technologies, Inc., Santa Clara, CA, USA) and sampled at 0.1 Hz. For the overall back pain data, the data collected during the final minute of baseline period were averaged and used to represent the ‘pre-intervention’ response, and the data from the last one minute of the intervention were averaged and used as the post-intervention data. These data were used to evaluate the impact of each intervention on perceived back pain. All data are reported as mean and standard deviations (±SD). No statistical analysis of these data has been completed; all results are descriptive for exploratory purposes.

## 3. Results

### 3.1. Control Condition

**Figure 2:**
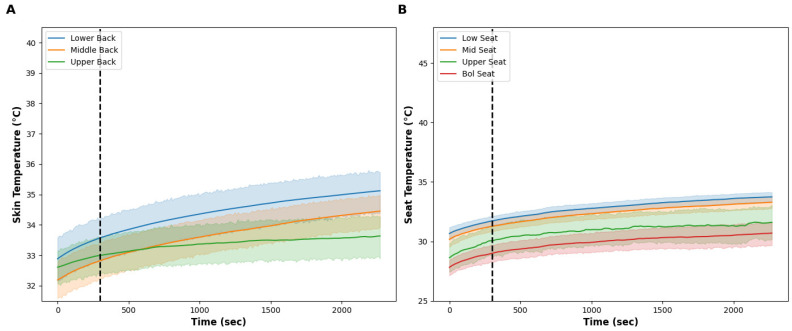
(**A**) shows the skin temperature at the low (blue line), middle (orange line), and upper (green line) back during the control condition. The vertical dashed line shows the onset of the test intervention. (**B**) shows the seat surface temperature at the low (blue line), middle (orange line), upper (green line), and bolster (red line) area of the seat during the control condition. The vertical dashed line shows the onset of the test intervention. Note the slight increase in skin and surface temperature from impaired heat dissipation due to the seat, even though the thermal effectors were turned off. The solid lines show the mean and the shaded areas are the standard deviation.

**Figure 3:**
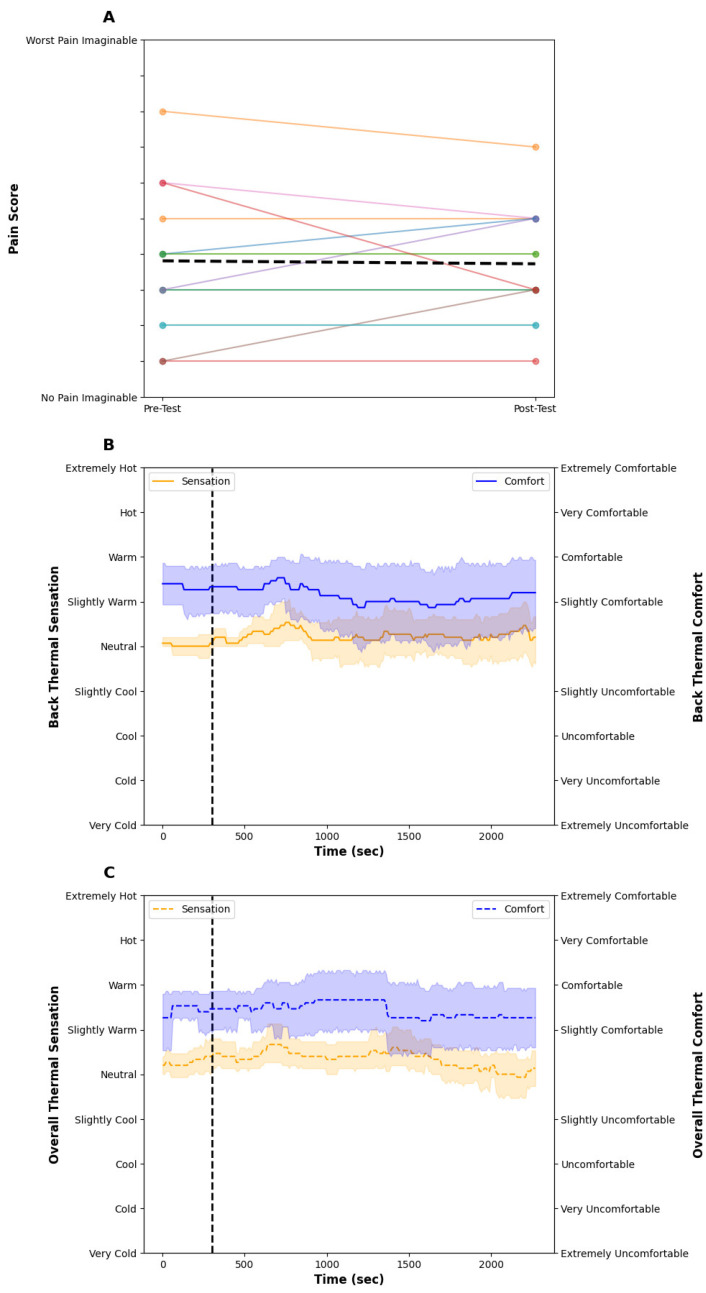
(**A**) shows the individual pre- and post-pain scores (colored lines) and the dashed black line indicates the mean group response. (**B**) shows the back thermal sensation (orange) and thermal comfort score (blue) during the control condition. The vertical dashed line shows the onset of the test intervention. (**C**) shows the overall thermal sensation (orange) and thermal comfort score (blue) during the control condition. The vertical dashed line shows the onset of the test intervention. The individual pain response shows high variability from sitting in a car seat. Thermal comfort and sensation remained stable and ‘slightly comfortable’ and ‘neutral’ throughout the test, respectively. The solid lines show the mean and the shaded areas are the SD.

The room temperature was 22.0 ± 1.4 °C throughout the test. Figure 2 illustrates the skin temperature (A) and seat surface temperature (B), and Figure 3 contains the subjective pain scores (A), back thermal sensation (orange) and thermal comfort (blue) scores (B), and overall thermal sensation (orange) and thermal comfort (blue) scores (C). The seat surface temperature of the low, middle, and upper regions slightly increased but remained stable over time. The seat surface temperature was about 2 °C lower than skin temperature. The skin temperature of the lower and mid back increased by ~1.5 °C from the start of the baseline to the end of the 33-min test; however, there were no noticeable differences between each skin temperature at the lower, middle, and upper back. The average subjective pain score was 3.8 ± 1.9 and this did not change, pre- or post-test. Examining the individual data, some participants’ pain was exacerbated from sitting, whereas other data demonstrate a decrease or no change. The back and overall thermal sensation remained at ‘neutral’ throughout the test and their thermal comfort score was ‘slightly comfortable’ throughout. After the 5-min baseline, participants felt the seat at all nine locations represented in the upper right of Figure 1 felt ‘neutral’ throughout the test.

### 3.2. Localized Therapy

**Figure 4:**
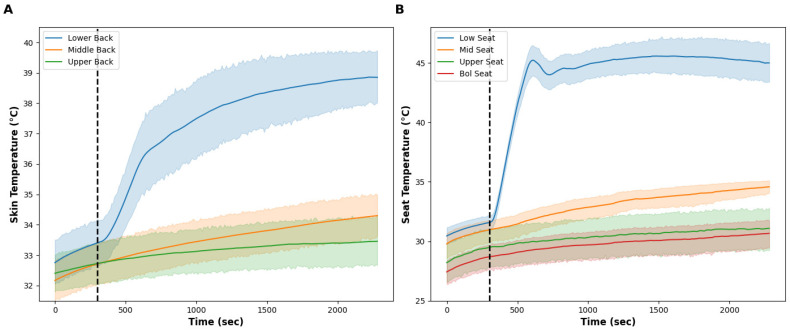
(**A**) shows the skin temperature at the low (blue line), middle (orange line), and upper (green line) back during the localized condition. The vertical dashed line shows the onset of the test intervention. (**B**) shows the seat surface temperature at the low (blue line), middle (orange line), upper (green line), and bolster (red line) area of the seat during the control condition. The vertical dashed line shows the onset of the test intervention. Only the skin temperature of the lower back was impacted by increasing the temperature of the lower portion of the seat back. The solid lines show the mean and the shaded areas are the standard deviation.

**Figure 5:**
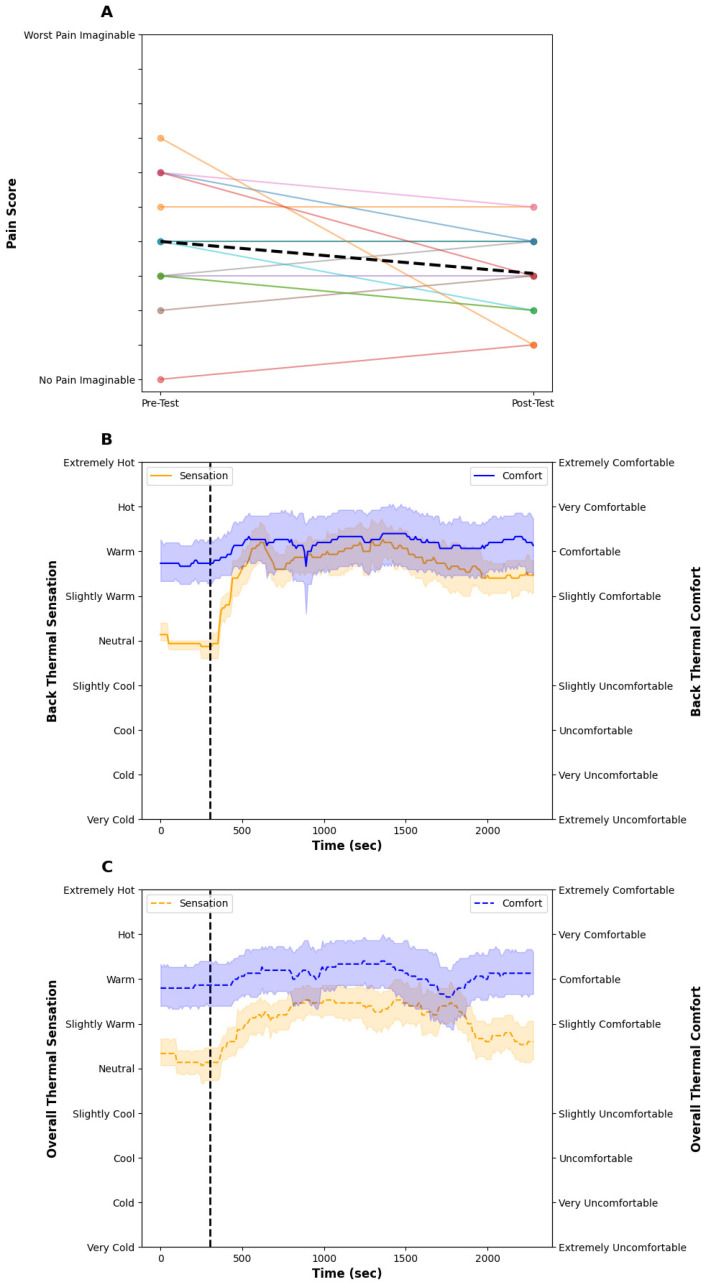
(**A**) shows the individual pre and post-pain scores (colored lines) and the dashed black line indicates the mean group response. (**B**) shows the back thermal sensation (orange) and thermal comfort score (blue) during the control condition. The vertical dashed line shows the onset of the test intervention. (**C**) shows the overall thermal sensation (orange) and thermal comfort score (blue) during the control condition. The vertical dashed line shows the onset of the test intervention. The individual pain data shows most participants having a reduction in subjective pain from the heating intervention. Upon turning the thermal effectors on, local and overall thermal sensation increased to ‘warm’ and ‘slightly warm’, respectively, whereas thermal comfort was not impacted. The solid lines show the mean and the shaded areas are the SD.

The room temperature was 22.7 ± 1.4 °C for the localized condition. Figure 4 illustrates the skin temperature (A) and seat surface temperature (B), and Figure 5 contains the subjective pain scores (A), back (B) and overall (C) thermal sensation (orange) and thermal comfort (blue) scores. After the 5-min baseline period, the surface temperature of the lower region of the seat increased to ~45 °C within ~7 min of turning the thermal effectors on and remained stable at ~45 °C throughout the test. At the end of the 33-min thermal intervention, the seat surface temperature of the low region (45.2 ± 3.2 °C) was higher than the mid (34.4 ± 1.3 °C), upper (31.0 ± 3.4 °C), and bolster regions (30.6 ± 2.4 °C) of the seat. The skin temperature of the lower back started at 33.1 ± 1.5 °C and increased to 38.8 ± 1.8 °C by the end of the test. The skin temperatures of the mid and upper back were stable at approximately 33 °C during the test. The average subjective pain scores decreased from 4.0 ± 1.9 at baseline and decreased to 3.1 ± 1.3 post-test. The back thermal sensation started at ‘neutral’ and then increased to ‘warm’ when the seat started heating. Similarly, the overall thermal sensation followed the same trend as the back thermal sensation, reaching a stable ‘slightly warm’ sensation throughout the test. Both the back and overall thermal comfort were rated as ‘comfortable’ throughout. Following the baseline, participants indicated that their lower back felt warm within ~60 s of turning the thermal effectors on. This warm sensation spread to the middle back after ~5 min and remained throughout the test.

## 4. Discussion

Automotive seat heating technology has been in production for several decades, and although not a standard feature in all vehicles, the adoption rate is increasing year-on-year across all trim levels and expanding to all seating locations within the vehicle. With the transition to battery electric vehicles, the need to create microclimates in the vehicle to provide thermal comfort to the occupant rather than conditioning the entire cabin will see microclimate technologies become more commonplace to extend the battery range of electric vehicles [20]. Although originally designed for thermal comfort, there is anecdotal evidence of occupants using their heated seats to help relieve back pain, soothe sore muscles, and even alleviate pain from menstrual cramps. This is unsurprising, given that humans have been using thermal modalities for managing pain and injuries, and facilitating recovery from exercise, for several centuries, dating back to Hippocrates [24]. This study investigated whether the heating technology in an automotive seat can reduce lower back pain within the confines of a common drive time in North America, Asia, and Europe [19,25,26]. The thermal profiles of both the seat surface temperature and skin temperature of the back indicate that we were successfully able to provide heat only to the desired location (i.e., the lower back) which facilitated a meaningful decrease in subjective lower back pain.

For the ‘localized’ condition, the aim was to increase the seat surface—and therefore skin—temperature only in the area of the body experiencing pain (i.e., the lower back). The technology within the seat allowed us to control and increase the temperature of only the lower back region within the seat back. During the 33-min intervention, the surface temperature of the lower third of the seat increased to ~45 °C, whilst the middle and upper seat surface temperatures remained below ~34 °C. Localized heating caused a reduction in subjective back pain by 1 unit on a 0 to 10 scale, which is considered a moderate effect [27]. There was high individual variability in subjective back pain data; it is noticeable that the individual who had the highest subjective pain response at the start of the protocol had the largest reduction in back pain. It has been suggested that the change in pain intensity that is meaningful to patients increases as the severity of their baseline pain increases. Therefore, someone experiencing moderate pain will find a smaller change in the subjective pain score as meaningful [28]. Our sample size is too small, with only five people with each type (acute, sub-acute, chronic) of lower back pain, to explore this phenomenon in greater detail, but there appears to be a trend for improved pain with the local heating—but improvements could be made for greater applicability to all. While examining the pain-alleviating impact of localized heating was our primary goal, the data indicate a potentially battery-saving potential in thermal comfort as well.

This ability to only provide heat to the lower back allowed for occupants to remain thermally comfortable and not become overheated (see Figure 5C) which might have occurred if the entire seat back was heated to such temperatures, within a neutral environment. Lower back skin temperature averaged ~39 °C and participants reported this to be ‘warm’ and ‘comfortable’. While this temperature is below the typical therapeutic heat level of 40 °C [7,15], we demonstrated a one-unit decrease in subjective pain, an impact equal to common over-the-counter remedies (NSAIDs) [27]. This is important for the ability of the occupant to use this thermal profile in warmer conditions, as this amount of heat applied to the skin for 33 min did not impact overall thermal comfort. To optimize battery-saving potential in battery electric vehicles, providing heat to other areas of the body may provide better thermal comfort results. For example, the warm thermal sensitivity of the buttocks and back of the neck are greater compared to the lower back, and this may provide more thermal-comfort benefits within microclimate heating [29]; however, as the purpose of this study was to examine the impact of local heating on LBP, this question remains to be tested.

The control condition provided insightful information about the increases in seat and skin surface temperature from sitting on a seat in the absence of any thermal technologies. Skin temperature increased across the back by ~1.5 °C during the 33-min intervention. The rise in skin temperature occurred due to impaired heat loss from the body caused by the seat, and thus the skin temperature slowly rose. Because, in the control condition, the seat did not produce heat, the rise was small but stable during sitting due to consistent metabolic activity and the inability to dissipate the heat produced fully. This rise in skin temperature is in line with some of the limited data available in similar studies [30]. The seat surface temperature rose very slightly but was ~2 °C below skin temperature throughout the test. The control seat did not affect the subjective pain scores. The individual scores in the pain data might suggest that for some people sitting helped relieve their pain, whilst for some participants the prolonged sitting aggravated their back pain and some scores increased slightly.

Several car manufacturers limit the seat surface temperature to 43–45 °C for fear of burning occupants [31,32]. Our data suggest that this is too conservative, especially because skin temperature (38.8 ± 1.8 °C) remained below the pain threshold (43 °C) and the threshold for burn injury (45 °C) [31,32,33], from only wearing a cotton T-shirt as insulation (clo ~0.15). Indeed, these guidelines were created from direct contact with hard, solid surfaces such as plastics, ceramics, and metals [32]. The thermal conductivity (k) for these solids (metals, k = 45–200 W/(m·K); glass, stone, plastics, k = 0.12–2.5 W/(m·K)) is higher than materials used in the seat trim (leather, k = 0.1–2.5 W/(m·K); polyester, k = 0.04–0.06 W/(m·K)). Additionally, there are several layers between the heating element and the skin, such as a foam layer, a layer of leather or polyester, and more than likely at least a t-shirt, if not additional layers of clothing, such as an undershirt, sweater, etc. Furthermore, the time spent at this skin temperature is well within the temperature–time limit guidelines recently published [31]. This report indicates that the temperature–time limit for first-degree burns for 45 °C is 2 h [31]. The time that the lower region of the seat surface spent above 45 °C was 30 min, with participants perceiving it as thermally warm and thermally comfortable. It is important to note that while the seat surface temperature reached 45 °C, the skin temperature on the back was ~6 °C below this limit. It is possible that if we had increased the seat surface temperature, and thus the skin temperature, the impact on reported back pain might have been greater. To support this, participants often reported that they often wanted more heat than what was provided, which is supported by none of the participants rating the temperature less than “slightly comfortable”. There seems to be room for improvement with our current approach.

Despite heat being used to manage pain, the precise mechanism(s) responsible for the analgesic effect of temperature remains unclear. Previous studies provide limited physiological data and are typically limited to device-level temperature. Additionally, it is unclear what skin temperatures are known to be “therapeutic”. The consensus is that a skin temperature > 40 °C is therapeutic, [7,15]; however, we demonstrate pain reduction with a skin temperature < 40 °C. The activation of TRPV-1 is thought to desensitize pain receptors [11,12] and these receptors are activated at temperatures > 43 °C. The skin temperature of the lower back averaged ~39 °C which would be too low to activate TRPV-1 receptors, but would have activated TRPV-3 and TRPV-4 receptors [10]. Whether these receptors also play a role in pain management is unclear. Other impacts of local heating involve thermal analgesia, gate control theory, or an increase in blood flow through the relaxation of smooth muscle from local factors, such as histamine, prostaglandin, and bradykinin, and central factors including a withdrawal of sympathetic tone from the stimulation of thermoreceptors [6,13]. It would be interesting to see if we could have had a stronger impact on pain management if we had increased the seat surface and thus skin temperature in our study; however, we wanted to be within the guidelines that several car manufacturers follow [31]. The upper limits of this are unknown but some of our participants mentioned they would have liked it to be hotter, although this was not systematically measured/recorded. It would be interesting to capture the skin temperature efficacy range and if there is a temperature-response curve. Finally, as the ideal use case would be to receive this ‘therapy’ each time an occupant is in their vehicle, this may unlock additional compounding effects of repeated bouts of local heating, as there is limited evidence of local heating benefits lasting longer than 3 months [15].

### Methodological Considerations

As this was an exploratory study of acute, sub-acute, and chronic back pain conditions, we did not recruit sufficient participants to conduct statistical analyses, as this study is underpowered. Nevertheless, we demonstrate from the individual results presented in Figure 3 and Figure 5 that a single bout of local heating provided to the lower back for 33 min reduced subjective lower back pain in the majority of individuals. While the study design was to investigate the ability to reduce lower back pain from an automotive seat during a single commute, drivers and passengers would be able to receive localized heating any time they are in the vehicle, potentially unlocking a compounding effect of repeated bouts of thermal therapy; however, at this time that remains unknown. Finally, as we followed recent automotive guidelines [31], it is unknown if hotter temperatures would unlock further pain-alleviating effects. Indeed, as many participants indicated they wanted hotter temperatures and none reported any thermal pain, increases in seat surface temperature may be beneficial.

## 5. Conclusions

This pilot study examined the effect of locally heating the lower back from an automotive seat for managing people’s lower back pain within the time constraints of a typical commute. A control condition provided an overview of the temperature changes from just sitting on a seat, and this had no impact on subjective back pain. Subjective back pain was reduced when local heating was applied to the lower back only. Our exploratory study indicated that the heating technology in automotive seats can help manage lower back complaints in people who experience low back pain, within time constraints of a typical commute, provided in a fashion that could seamlessly be incorporated into a driver’s typical day.

## 6. Patents

U.S. and international patent applications: US20240108495A1 [34], WO2024072647A1 [35].

## Data Availability

The datasets for this study are not publicly available for proprietary reasons.
